# The impact of high-sugar diets on central nervous system disorders: mechanisms, pathogenesis, and dietary implication

**DOI:** 10.1080/07853890.2025.2561789

**Published:** 2025-09-18

**Authors:** Fang Li, Qinghua Luo, Tinghao Guo, Shixian Zhou, Zhijuan Cheng, Hanqing Pan, Jianglong Tu

**Affiliations:** ^a^ Department of Neurology, The Second Affiliated Hospital of Nanchang University, Nanchang, Jiangxi, China; ^b^ Institute of Neuroscience, Nanchang University, Nanchang, Jiangxi, China; ^c^ Jiangxi Medical College, Nanchang University, Nanchang, Jiangxi, China; ^d^ Jiangxi Provincial Key Laboratory of Neurological Diseases, Nanchang University, Nanchang, Jiangxi, China; ^e^ JXHC Key Laboratory of Neurological Medicine, Nanchang University, Nanchang, Jiangxi, China

**Keywords:** High-sugar diet, Western diet, metabolic, central nervous system, mechanisms, neurodegenerative

## Abstract

**Background:**

Excessive sugar consumption has paralleled the global rise in obesity, type 2 diabetes mellitus (T2DM), and related metabolic disorders. High-sugar diets directly contribute to weight gain, insulin resistance, and chronic hyperglycemia, which drive cardiovascular complications and systemic inflammation through advanced glycation end products (AGEs) and oxidative stress. Emerging evidence highlights their critical role in the pathogenesis of central nervous system (CNS) disorders, including Alzheimer’s disease, Parkinson’s disease, and stroke, likely mediated through obesity-associated chronic inflammation, T2DM-driven blood-brain barrier dysfunction, and neuroinflammation.

**Rationale and aim of the study:**

This review explores the impact of high-sugar diets on CNS diseases, focusing on the mechanisms involved, such as neuroinflammation, oxidative stress, insulin resistance, and altered neurotransmission. Methods: For this purpose, databases, such as PubMed, Medline, and PubMed Central (PMC) have been searched.

**Results:**

Accumulating evidence underscores the detrimental impact of high-sugar diets, particularly those high in glucose and fructose, on CNS diseases. These dietary patterns are linked to the exacerbation of various CNS diseases through multiple pathways. After analyzing the current literature, we can develop targeted dietary interventions aimed at reducing the burden of CNS diseases associated with high-sugar diets.

**Discussion and conclusion:**

This paper emphasizes the importance of adopting nutritional approaches that address both metabolic and neurological health to combat the growing burden of CNS diseases linked to high-sugar consumption.

## 1. Introduction

High-sugar diets (HSD), which are a hallmark of the Western diet, have been strongly linked to a wide array of serious health issues, including obesity, insulin resistance, diabetes, non-alcoholic fatty liver disease, mental disorders, and central nervous system (CNS) diseases [1–5]. According to a recent report by the World Health Organization (WHO) and the Food and Agriculture Organization (FAO) of the United Nations, sugars include monosaccharides, disaccharides, polyols, and free sugars [6–8]. Free sugars refer to all monosaccharides and disaccharides added to foods by manufacturers, cooks, or consumers, as well as naturally occurring sugars in honey, syrups, and fruit juice. WHO strongly recommends reducing free sugars intake to <10% of total energy, both in children and adults [9]. Despite these recommendations, the global overconsumption of free sugars has reached alarming levels, driven by the increased use of sucrose and high-fructose corn syrup in processed foods and beverages [8]. For instance, overconsumption rates have reached 61% in the UK, 41% in France, and 49.5% in the US [10]. Notably, the U.S. data expose significant gender-specific susceptibility, with males exhibiting 1.45-fold higher overconsumption rates than females (59.3 *vs.* 40.7%) [11]. Emerging evidence suggests that sex-specific disparities in hepatic fructose and sucrose metabolism, mediated by estrogen-regulated enzymes, such as fructokinase and gluconeogenic regulators [12,13], may contribute to the heightened risk of metabolic and neurological disorders in women. Preclinical studies demonstrate that female rodents exhibit exacerbated hepatic lipogenesis and systemic inflammation under high-sugar diets compared to males [14–17], paralleling clinical observations of increased female susceptibility to Alzheimer’s Disease (AD) and neuroinflammation-linked pathologies [18–21]. Beyond metabolic and neurodegenerative pathologies, high-sugar diets and resultant hyperglycemia significantly impact mental health. Epidemiological studies indicate that individuals with diabetes exhibit poorer self-rated health (SRH), a robust predictor of morbidity and mortality [22]. Critically, diabetes moderates the association between advancing age and declining SRH, suggesting accelerated health deterioration trajectories [23]. Furthermore, compelling evidence links chronic hyperglycemia and insulin resistance, hallmarks of high-sugar diet dysregulation, to an increased risk of anxiety and depression [24–28]. This intersection of metabolic dysregulation, adverse mental health outcomes (like anxiety and depression), perceived health status decline, and brain aging underscores the profound and multifaceted neurological burden imposed by chronic hyperglycemia.

Although individual susceptibility to metabolic disturbances may differ [29], excessive sugar consumption, defined here as intake more than recommended thresholds, is broadly associated with poor health across diverse cohorts [30–33]. In recent years, the impact of high-sugar diets on the pathogenesis of various CNS diseases has garnered significant interest among scientists, particularly regarding ischemic stroke, cerebral atherosclerosis, multiple sclerosis, neuromyelitis optica, Alzheimer’s disease, and Parkinson’s disease. Given the complex and multifaceted nature of CNS disorders, it is crucial to design dietary strategies that consider the intricate mechanisms by which high-sugar diets contribute to these CNS disorders (Table 1). This paper aims to provide a comprehensive overview of the impact of high-sugar diets on CNS diseases, examining the underlying mechanisms and the crosstalk between metabolic and neurodegenerative processes.

**Table 1.** Summary of rodent model studies on dietary interventions.

Rodent model	Sugar/fat composition	Treatment duration	Neurometabolic/behavioral outcomes
Global cerebral ischemia(2-vessel occlusion)	20% fructose water	11 weeks	The HFD group rats exhibited worse neurological performance at 6 h post-ischemia [34].
Experimental autoimmune encephalomyelitis in mice	Caffeine-free high sucrose cola beverages (HSCB)	8 weeks before EAE induction and throughout the duration of disease progression.	HSCBs exacerbated experimental autoimmune encephalomyelitis pathogenesis in mice *via* microbiota-dependent mechanisms, specifically by modifying microbial community structure and enhancing Th17 cell populations [35].
Experimental autoimmune encephalomyelitis in mouse	20% glucose water	2 weeks before EAE induction and throughout the duration of disease progression.	High glucose intake aggravated autoimmunity in colitis and EAE mouse models by driving Th17 cell differentiation *via* ROS-dependent activation of latent TGF-β in T cells [36].
APP/PS1 double transgenic mice	10% sucrose in water	25 weeks	Long term consumption of sucrose-sweetened water causes more weight gain, induces insulin resistance, and exacerbates AD-like cognitive impairment and cerebral amyloid [37].
3xTg-AD mice	20% sucrose water	12 weeks	High sucrose intake exacerbates Aβ deposition and tau phosphorylation in AD model mice by activating the mTOR pathway [38].

## 2. Mechanisms linking high-sugar diets to CNS pathogenesis

**Figure 1.**
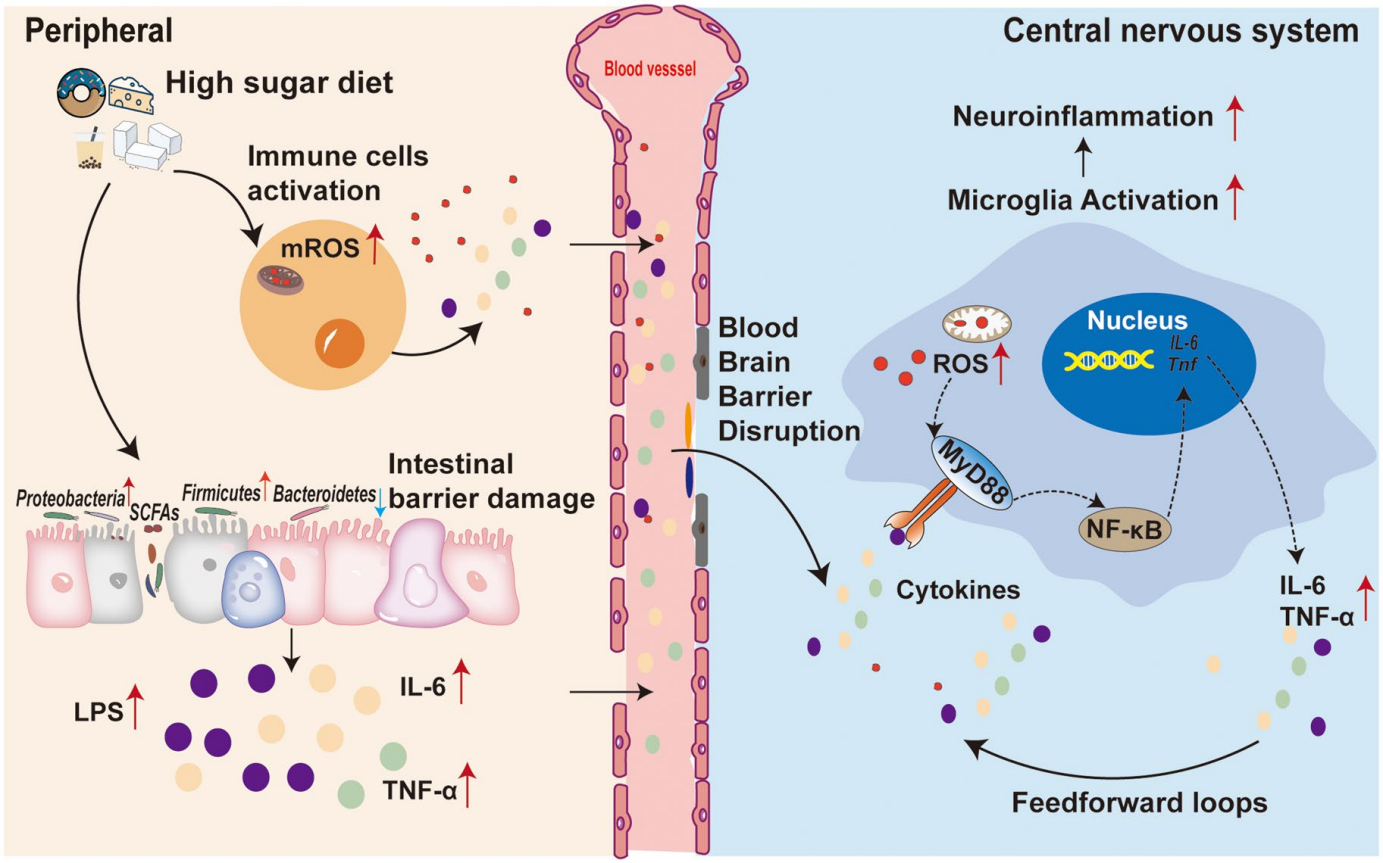
Mechanisms linking high-sugar diets to CNS pathogenesis. Peripherally, high-sugar diets both activates immune cells, elevating mitochondrial reactive oxygen species (mROS), and disrupts gut microbiota homeostasis, damaging the intestinal epithelial barrier. These combined effects synergize to enable systemic leakage of bacterial endotoxins (e.g. LPS) and pro-inflammatory cytokines (IL-6, TNF-α), amplifying peripheral immune activation and oxidative stress. Circulating inflammatory mediators compromise blood-brain barrier (BBB) integrity by disrupting endothelial tight junctions and increasing ROS levels. In the CNS, infiltrating LPS and cytokines activate microglia, initiating MYD88-dependent signaling, further cytokine/ROS release, and self-reinforcing feedforward loops that exacerbate neuroinflammation.

HSD are increasingly recognized as a significant factor in CNS pathogenesis, contributing to various neurological and neurodegenerative disorders. Here’s an in-depth look at the possible mechanisms linking high-sugar diets to CNS pathogenesis (Figure 1).

### 2.1. Systemic inflammation

HSD have been shown to trigger metabolic disturbances and increase the production of inflammatory mediators and pro-inflammatory cytokines in various tissues, which leads to insulin resistance and low-grade chronic inflammation [39–42]. Overconsumption of dietary sugars leads to insulin resistance and chronic low-grade inflammation, driven by inflammatory factors, such as nuclear factor kappa B (NF-κB) and tumor necrosis factor alpha (TNF-*α*) secreted by the liver and adipose tissue [3,43]. Moreover, high-sugar stimulation activates various immune cells, including monocytes, dendritic cells, and macrophages, which subsequently release pro-inflammatory cytokines, such as IL-6 and TNF-*α* [44]. Similarly, Jone Nicholas et al. demonstrated that mice fed a 10% mixture of glucose and fructose for two weeks had significantly higher serum levels of IL-6 and TNF-*α* [45]. Additionally, TNF-*α* released from dendritic cells induced by high-fructose stress can further trigger IFN-γ secreted by T cells [46].

The CNS is particularly vulnerable to harm from systemic inflammation. Cytokines, chemokines, and soluble mediators associated with damage from systemic inflammation can enter the CNS through the bloodstream or *via* the compromised blood-brain barrier (BBB), leading to neuronal and glial cell dysfunction and further neuroinflammation [47,48]. For instance, in models of LPS-induced systemic inflammation, significant upregulation of inflammatory genes like *Il-1β*, *Il-6*, *Tlr2*, and *Tlr4* in the cortex occurs within hours of inflammation onset [49,50]. Therefore, the association between high sugar intake and increased risk of CNS disorders may be mediated in part by low-grade chronic inflammation, which disrupts brain homeostasis and affects brain structure and function.

### 2.2. Microglial activation

Microglia, the resident immune cells of the CNS, serve as crucial regulators of neural homeostasis and neuroinflammatory responses. In their resting state, these cells display a highly ramified morphology with extensive, motile processes that constantly survey the CNS microenvironment [51]. Upon encountering pathological stimuli, such as damage-associated molecular patterns (DAMPs), pathogens, or protein aggregates, microglia undergo rapid activation characterized by profound morphological alterations, cell body hypertrophy, process retraction, and transformation into an amoeboid phenotype changes that enhance their migratory and phagocytic capacities [52,53]. Malvaso et al. [54] established a standardized scoring system to quantify these activation-associated morphological changes, providing researchers with a valuable semi-quantitative tool for assessing neuroinflammatory states across various pathological conditions. The activation process involves distinct morphological and functional transitions. Microglia develop polarized structures with leading pseudopodia for targeted chemotaxis, while simultaneously modulating surface marker expression (e.g. decreased CX3CR1 and increased Iba1/CD68) [55,56]. These changes reflect their functional shift from surveillance to active immune modulation. Notably, activation states exhibit phenotype-specific morphology, the pro-inflammatory M1 subtype adopts a spherical shape and secretes inflammatory mediators, while the reparative M2 phenotype retains partial ramification and promotes tissue repair [57]. This remarkable plasticity allows microglia to precisely balance neuroprotective and neurotoxic effects in the CNS [58,59]. For instance, in neurodegenerative disorders, such as AD [60] and PD [61], microglia actively clear misfolded proteins (e.g. pTau, *α*-synuclein), apoptotic cells, A*β* deposits (through phagocytosis and compaction), and damaged synapses. These neuroprotective activities may initially attenuate pathological progression during early disease stages. However, the efficiency of this neuroprotective function frequently declines with aging, primarily attributable to increased oxidative stress and mitochondrial dysfunction [54]. The diminished clearance capacity of microglia leads to accumulation of protein debris and sustained low-grade inflammation (inflammasome activation). In brain regions with concentrated neurodegeneration (potentially compounded by chronic hypoxia), microglia may lose their homeostatic functions, becoming either functionally exhausted/dystrophic or excessively activated and neurotoxic [54,56,62]. Consequently, through these dual mechanisms, impaired clearance capability and exaggerated inflammatory responses, microglia ultimately exacerbate neurodegeneration and synaptic loss [54]. Beyond neurodegenerative conditions, microglia also play significant roles in stroke, traumatic brain injury, and multiple sclerosis by modulating inflammatory responses and promoting neural repair [63–66]. HSD have been shown to activate microglia, which significantly increases oxidative and inflammatory stress in the brain [67,68]. High glucose levels can directly activate microglia, causing them to adopt an amoeboid morphology and produce pro-inflammatory cytokines, such as TNF-*α*, IL-1*β*, and IL-6, and upregulation of surface markers like CD11b and MHC-II [68–72]. Notably, under these hyperglycemic conditions, heat shock protein 70 (HSP70), a multifunctional chaperone induced by cellular stress, shows significant upregulation, which may serve as an adaptive mechanism to counteract excessive inflammation and restore microglial homeostasis [68]. Similar to glucose, a high-fructose diet has also been shown to modulate the inflammatory profile of the brain. Research has demonstrated that such a diet can increase the expression of Glut5 (a fructose transporter) and pro-inflammatory cytokines like TNF-*α*, particularly in the hippocampus, a critical region for memory and learning [73–75]. This hippocampal inflammation primarily manifests as retrograde amnesia (RA), characterized not only by an inability to form new long-term memories but also by a failure to recall information from events learned before the hippocampal damage [76], it is important to note, however, that retrograde amnesia frequently coexists with anterograde amnesia in hippocampal pathologies, reflecting the region’s integral role in both memory encoding and retrieval. Key impairments include deficits in retrieving previously acquired spatial memories (evidenced by impaired performance in established Morris water maze tasks) [77]. These RA manifestations are mediated through synaptic mechanisms involving downregulated BDNF and Arg3.1 synaptic dysregulation [78–81]. This high-fructose diet-induced pro-inflammatory effects are primarily mediated by signaling pathways, such as mitogen-activated protein kinases (MAPKs), phosphoinositide 3-kinase/Akt (PI3K/Akt), and NF-κB [82]. Additionally, high fructose consumption also leads to the buildup of AGEs in plasma and tissues [83–85]. AGEs bind to their receptor RAGE on microglia, activating inflammatory pathways, particularly NF-κB, which further amplifies microglial activation and inflammation in the CNS [86,87]. In essence, as described in Section 3.1, HSD provoke systemic and cerebral inflammation, which then secondary activates microglia, resulting in the release of additional pro-inflammatory cytokines and reactive oxygen species (ROS) that perpetuate a cycle of neuroinflammation, further exacerbating CNS pathology [4,49,88]. Microglia are widely recognized as key players in the progression of various neurological disorders, including PD. Specifically, they contribute to dopaminergic neurodegeneration through sustained NLRP3 inflammasome activation [89], which elevates IL-1*β* and tumor necrosis TNF-*α* production [90], thereby directly mediating neuronal loss [91]. Additionally, *α*-Syn preformed fibrils (PFFs) and the excessive ROS and nitrogen species (RNS) further drive microglial activation, exacerbating neuroinflammation and oxidative stress [92,93]. Chronic activation of microglia due to high-sugar consumption (fasting blood glucose >25 mM) leads to sustained neuroinflammation and oxidative stress through metabolic reprogramming [94]. This process involves a Warburg-like glycolytic shift [95] with excessive production of pro-inflammatory cytokines (IL-1*β*, IL-6) and ROS [94,96]. These factors may synergistically contribute to AD progression by amyloid-*β* aggregation and compromising blood-brain barrier integrity [97]. By continuously driving inflammation and oxidative damage, high-sugar diets contribute directly and indirectly to the pathological processes affecting the CNS.

### 2.3. Blood-brain barrier (BBB) disruption

The blood-brain barrier (BBB) is a selective, protective barrier formed by endothelial cells, pericytes, and astrocytes that shields the brain from harmful substances circulating in the blood while allowing the passage of essential nutrients [98]. In healthy conditions, glucose transport through the BBB is tightly regulated by specific transporters, such as sodium-independent glucose transporters (GLUTs) [99]. However, HSD are increasingly implicated in compromising BBB integrity, leading to heightened permeability and allowing harmful agents, immune cells, and inflammatory mediators to infiltrate the CNS [100].

HSD, particularly those rich in glucose and fructose, cause rapid spikes in the blood glucose levels [101], which can contribute to endothelial dysfunction in the BBB. Hyperglycemia reduces the production of endothelial nitric oxide (NO), a key molecule responsible for maintaining BBB integrity [102]. Furthermore, HSD increase pro-inflammatory cytokines like IL-6, TNF-*α*, and IL-1*β* in circulation (as described in Section 3.1). These inflammatory mediators can disrupt tight junction proteins (TJPs) like claudin-5 and occludin, which are essential for preserving BBB integrity [103]. For example, in streptozotocin-induced diabetic rats, BBB permeability was enhanced by a decrease in tight junction proteins (i.e. occludin and ZO-1) and an increase in matrix metalloproteinase activity [104].

On the other hand, a high sucrose diet increases the production of AGEs in endothelial cells and microglia, causing astrocytes to enter a proinflammatory state that disrupts the selective permeability of the BBB [5]. In addition to the inflammatory effects, elevated glucose and fructose levels promote oxidative stress, as these sugars increase the production of ROS (described in detail in the following Section 3.5), which degrade tight junction proteins and lead to the breakdown of the BBB [105,106]. ROS not only damage the structural integrity of the BBB directly but also activate microglia and astrocytes, resulting in the release of additional pro-inflammatory cytokines, which perpetuate a damaging feedback loop, further exacerbating BBB disruption and CNS damage [107]. Moreover, hyperglycemia-related complications, including cerebral ischemia, hypertension, and hyperosmolality, further exacerbate BBB dysfunction, allowing typically restricted biomolecules to infiltrate brain parenchyma [108]. In conclusion, HSD play a significant role in the progression of CNS diseases by disrupting the integrity of the BBB. The breakdown of the BBB allows harmful substances to enter the brain, initiating a cascade of neuroinflammatory responses and oxidative stress, which are further linked to reduced cerebral blood flow and increased susceptibility to CNS disorders, such as AD, PD, and stroke [109].

### 2.4. Gut-brain axis modulation

The gut-brain axis is a complex, bidirectional communication network linking the gastrointestinal (GI) tract and the CNS, influencing brain function and behavior through neural, endocrine, immune, and metabolic mechanisms [110]. HSD have been shown to negatively affect gut health, which can disrupt the gut-brain axis and contribute to CNS pathogenesis [35,111–113]. Research in rodents has shown that HSD cause significant shifts in the composition of the gut microbiota, with an increase in harmful bacteria, such as *Proteobacteria*, *Firmicutes*, and a reduction in beneficial bacteria like *Bacteroidetes* [112,114,115]. This microbial imbalance, or gut dysbiosis, weakens the gut barrier, facilitating the translocation of harmful microorganisms and their metabolites into the bloodstream, thereby increasing systemic inflammation [116,117].

Glucose and fructose-enriched diets have been shown to impair intestinal barrier integrity through mechanisms, such as TLR4-mediated colonic inflammation [118], glucose transporter 2 (GLUT2)-mediated retrograde uptake of glucose into epithelial cells resulting in increased N-glycan biosynthesis [119], or downregulation of TJPs [117,120–122]. For instance, in diabetic mouse models, scavenging of intestinal flora by broad-spectrum antibiotics has been shown to reduce systemic lipopolysaccharide (LPS) levels and decrease intestinal permeability by upregulating TJPs like ZO-1 and occludin [123]. Increased gut permeability allows LPS and other endotoxins to enter the bloodstream, triggering systemic inflammation, which in turn contributes to neuroinflammation and CNS dysfunction [113].

The intestine is the largest immune organ and interfaces dietary components with the host [124]. HSD have been implicated in increasing intestinal inflammation, which can contribute to endotoxemia and tissue inflammation [125,126]. Dietary sugar-induced microbiota imbalance can disrupt immune-mediated protection from metabolic syndrome by altering populations of immune cells, such as reducing commensal Th17 cells and increasing certain bacterial families like *Erysipelotrichiaceae* [127]. Moreover, microbiota-derived metabolites, such as short-chain fatty acids (SCFAs) are crucial for maintaining gut barrier function and modulating immune responses. SCFAs, especially butyrate, have neuroprotective effects by maintaining BBB integrity and suppressing neuroinflammation [128]. However, Western diets, which are low in fiber, reduce SCFAs production by limiting fiber-fermenting bacteria, thus depriving the brain of their protective benefits [129]. Additionally, the vagus nerve, a key mediator in the gut-brain axis, transmits inflammatory and neuroactive signals from the gut to the brain, exacerbating neuroinflammation, oxidative stress, and BBB dysfunction [130,131]. Collectively, these mechanisms highlight HSD may disrupt the gut-brain axis through neuroimmune, endocrine, and neural pathways, heightening susceptibility to CNS pathologies, including neurodegenerative diseases like AD and PD, as well as neurodevelopmental disorders [28].

### 2.5. Oxidative stress and cellular damage

Oxidative stress, resulting from an imbalance between ROS production and the brain’s ability to neutralize them, plays a crucial role in the development and progression of neurological disorders associated with high-sugar diets [36,132,133]. HSD, particularly those high in glucose and fructose, significantly boost ROS production, including superoxide, hydrogen peroxide, and hydroxyl radicals [132]. Elevated blood glucose levels lead to enhanced glycolysis and mitochondrial metabolism, which generates more ROS as byproducts [134]. Fructose, in particular, is metabolized in the liver through a pathway that generates a high amount of ROS and uric acid, a potent pro-inflammatory mediator [135].

The brain has evolved mechanisms to counteract ROS through antioxidant defenses. Enzymes like superoxide dismutase (SOD) and glutathione peroxidase, as well as molecules, such as trehalose, play crucial roles in scavenging ROS and protecting cells from oxidative damage [136]. However, HSD can overwhelm these antioxidant defenses, leading to oxidative stress that damages lipids, proteins, and DNA in neurons and glial cells [137,138]. For instance, animal studies have shown that high-sucrose diet can disrupt antioxidant systems by decreasing the activity of antioxidant enzymes like glutathione peroxidase and SOD in the hypothalamus [133].

One of the primary consequences of oxidative stress is lipid peroxidation. High levels of ROS attack polyunsaturated fatty acids in neuronal membranes, initiating a chain reaction that leads to the formation of lipid peroxides and aldehydes, such as malondialdehyde (MDA) [139]. Lipid peroxidation compromises membrane integrity, disrupts cellular signaling, and induces further oxidative stress, which can contribute to neuronal death and dysfunction [140]. Oxidative modification of proteins also can lead to the formation of oxidized proteins and AGEs, which accumulate as a result of high-sugar diets, further contribute to oxidative stress by promoting ROS production and inflammation. These modifications can impair protein function, promote protein aggregation, and trigger cellular apoptosis [141]. Similarly, ROS-induced DNA damage can cause mutations [142], genomic instability [143], and cellular senescence [144], all of which are linked to neurodegenerative diseases, such as AD and PD. Furthermore, HSD activate various stress response pathways, including the MAPK and NF-κB pathways [82], which further exacerbate oxidative stress and inflammation, and creating a vicious cycle of oxidative stress and inflammation that contributes to CNS pathogenesis.

Excessive intake of glucose and fructose also contribute to mitochondrial dysfunction. Mitochondria, responsible for ATP production through oxidative phosphorylation (OXPHOS) [145], become impaired due to increased ROS production [146]. High fructose-fed rat models exhibited mitochondrial metabolic reprogramming and dysfunction in podocytes [147]. Inefficiencies in OXPHOS can lead to the accumulation of ROS, which damages mitochondrial DNA and proteins, impairs ATP production, and disrupts cellular energy metabolism [148]. This mitochondrial dysfunction can result in neuronal cell death and contribute to various CNS diseases.

## 3. CNS disorders impacted by high-sugar diets

### 3.1. Ischemic stroke

Acute ischemic stroke is a leading cause of disability and death in adults, primarily resulting from atherosclerosis and thromboembolism [149]. Recent evidence suggests that high-sugar diets may exacerbate stroke risk through multiple pathways. For example, a large-scale cohort study demonstrated that individuals consuming ≥25% of daily calories from added sugars had a nearly 3-fold increased risk of cardiovascular mortality, including stroke, compared to those consuming <10%, concomitant with demonstrated significantly elevated rates of atherogenic dyslipidemia, sustained weight gain trajectories, and obesity prevalence [150]. Additionally, excess sugar intake may indirectly elevate stroke risk by promoting obesity and metabolic syndrome, as highlighted in the American Heart Association’s scientific statement [151]. Emerging evidence also links sugar-sweetened beverages to cerebrovascular dysfunction, though mechanistic insights remain under investigation [101]. High-sugar diets have been implicated in increasing the risk of metabolic disorders, such as obesity, diabetes, and hypertension, which are well-established risk factors for cerebrovascular diseases, including stroke [152–154].

Clinical studies have suggested a direct correlation between sugar intake in daily life and the incidence of cardiovascular and cerebrovascular events [155–157]. For instance, a predictive study from South Africa highlighted the potential benefits of a sugar-sweetened beverage tax, estimating it could save 550,000 stroke-related health-adjusted life years, and reduce the number of ∼85,000 new stroke cases and 13,000 existing cases over 20 years [158]. Similarly, a Swedish cohort study observed that consuming ≥2 servings of sugar sweetened beverages (SSBs) was associated with an increased risk of ischemic but not of hemorrhagic stroke [159]. A recent meta-analysis further supported this, revealing that higher consumption of SSBs significantly increased the risk of stroke (RR 1.12, 95% CI 1.03–1.23) and specifically increased the risk of ischemic stroke by 10% [160]. In line with human studies, experimental studies in animal models have demonstrated that long-term exposure to various forms of sugar, including fructose and artificial sweeteners like erythritol, acesulfame K, or rebaudioside A, significantly worsens cerebral ischemic injury, leading to increased infarct volumes, impaired neurobehavioral outcomes, reduced angiogenesis, and impaired endothelial progenitor cell function [161,162].

Mechanistically, high glucose diets may directly induce neuroinflammation and neuronal loss, which exacerbate ischemic stroke development. For example, a study demonstrated that a high-fructose diet increased neuronal loss and triggered significant astroglial and microglial immunoreactivity changes in the caudate putamen, resulting in worsened neurological performance in a cerebral ischemia rat model [34]. Hyperglycemia caused by a high-sugar diet is also implicated in ischemic stroke, with strong evidence showing that elevated glucose levels are independent predictors of larger infarct size, poorer clinical outcomes, and increased risk of mortality [163]. The odds ratio (OR) for poor outcomes in ischemic stroke associated with hyperglycemia has been reported as 3.1(95% CI, 2.3–4.3) [164], underscoring the critical role of high glucose levels in worsening stroke prognosis. Prolonged hyperglycemia contributes to microvascular and macrovascular pathologies, along with BBB permeability, promoting inflammation after stroke [165]. Recent findings by Abdul et al. showed that high glucose levels induce lipid peroxidation and ferroptosis in the brain endothelium of rats following ischemic stroke, exacerbating vascular degeneration during stroke recovery and impacting neurovascular remodeling [166]. This effect is possibly due to hyperglycemia enhancing neutrophil infiltration into the brain post-stroke and increasing the expression of lipocalin-2 (LCN2) in these cells. LCN2 promotes the accumulation of lipid peroxides by elevating intracellular ferrous iron levels, thereby exacerbating ferroptosis in key brain cells, particularly neurons. In addition, high-sugar-induced BBB breakdown has been linked with ischemic stroke progression [104,167,168]. High sugar diets increase ROS production, activating the NF-κB signaling pathway, which drives the expression of leukocyte adhesion molecules and cytokines, critical components in inflammation and BBB damage [169,170].

In summary, while the link between hyperglycemia and ischemic stroke is well established, the specific mechanisms through which high-sugar diets exacerbate stroke risk remain complex and multifaceted. More in-depth research is anticipated to clarify these mechanisms further and identify potential therapeutic targets.

### 3.2. Cerebral atherosclerosis

Cerebral atherosclerosis, a subtype of atherosclerosis that affects the arteries supplying the brain, is a significant contributor to ischemic stroke and other cerebrovascular diseases. The development of cerebral atherosclerosis involves the accumulation of fatty deposits or plaques, along with inflammation within the arterial walls, leading to their narrowing and stiffening. This process impedes cerebral blood flow, increasing the risk of cerebrovascular events like ischemic stroke [171], and resulting in subsequent neurological impairments [172,173]. While traditional risk factors for atherosclerosis, such as hypertension, hyperlipidemia, and smoking, are well recognized, recent evidence suggests that high-sugar diets may also play a crucial role in the pathogenesis of cerebral atherosclerosis [174]. A study on Mexican women revealed that those who consumed high amounts of sugary sodas had a 2.6% greater carotid intima-media thickness (IMT), an indicator of atherosclerosis, and were twice as likely to develop carotid atherosclerosis, with the risk particularly pronounced in older and postmenopausal women [175]. Similarly, Chun et al. [176] also found that sugar-sweetened sodas consumption was linked to the presence of coronary artery calcium, a marker of subclinical atherosclerosis that is closely related to IMT.

HSD contribute to the development of metabolic disorders, such as obesity [127,177], insulin resistance [177], and type 2 diabetes [178], which are well-established risk factors for atherosclerosis [179]. HSD lead to chronic hyperglycemia, which accelerates the formation of AGEs in the vascular system. AGEs are proteins or lipids that become glycated as a result of exposure to sugars, and they can induce oxidative stress and inflammation within the arterial walls [180]. This oxidative stress is a key driver in the endothelial dysfunction that precedes atherosclerosis [181]. Endothelial cells, which line the blood vessels, lose their ability to regulate vascular tone and maintain a barrier against harmful substances, resulting in increased permeability, leukocyte adhesion, and plaque formation [182]. Moreover, high glucose levels have been shown to stimulate the proliferation of vascular smooth muscle cells (VSMCs) and the deposition of extracellular matrix components [183], both of which contribute to plaque stability and the progression of atherosclerotic lesions. The excessive proliferation of VSMCs in response to hyperglycemia is mediated by the activation of the protein kinase C (PKC) pathway, which enhances the expression of pro-atherogenic molecules, such as endothelin-1 and transforming growth factor-beta (TGF-*β*) [184,185]. Furthermore, a high-sugar diet has been implicated in dyslipidemia, particularly by increasing levels of triglycerides and small, dense low-density lipoprotein (LDL) particles, both of which are highly atherogenic [186]. These lipid abnormalities further exacerbate the risk of cerebral atherosclerosis by promoting lipid deposition within the arterial walls and enhancing the inflammatory response.

In addition to these direct effects on the vascular system, high-sugar diets may exacerbate atherosclerosis by promoting systemic inflammation. Elevated levels of circulating glucose can activate the NF-κB signaling pathway, a key regulator of inflammatory responses. NF-κB activation leads to the increased production of pro-inflammatory cytokines, such as TNF-*α* and IL-6, which contribute to the inflammatory milieu within atherosclerotic plaques [187]. Chronic inflammation not only accelerates plaque formation but also destabilizes existing plaques, making them more prone to rupture and precipitating acute ischemic events [188]. Moreover, High-sugar diets may also influence cerebral atherosclerosis by modifying microglial activation [189]. In diabetic conditions marked by high-glucose environments, microglia in the brain tend to polarize toward an M2c-like state. This specific polarization has been implicated in the progression of cerebral atherosclerosis [190]. Animal studies have demonstrated that by inhibiting M2c-like microglial polarization can markedly reduce the severity of diabetes-related cerebral atherosclerosis [189]. In conclusion, high-sugar diets play a multifaceted role in the development and progression of cerebral atherosclerosis through metabolic dysregulation, oxidative stress, inflammation, and vascular damage.

### 3.3. Multiple sclerosis and neuromyelitis optica

Multiple sclerosis (MS), neuromyelitis optica (NMO), and autoimmune encephalitis (AE) are chronic autoimmune disorders of the CNS, characterized by neuroinflammation, demyelination (in MS/NMO), and/or neuronal dysfunction [191–193]. While MS primarily affects the brain and spinal cord [191], and NMO targets optic nerves and spinal cord [192], AE involves antibody-mediated attacks against neuronal surface or synaptic proteins (e.g. NMDA receptors, LGI1), leading to diverse neuropsychiatric symptoms, such as seizures, memory deficits, and behavioral changes [194]. Although the precise etiologies remain elusive, genetic susceptibility, environmental triggers, immune dysregulation, and dietary factors like high sugar intake are recognized contributors to disease progression and severity. Emerging evidence indicates that high-sugar diets exacerbate inflammatory and neurodegenerative processes across these disorders, including AE [195].

MS is pathologically distinguished by widespread inflammatory processes involving autoreactive T cells, B lymphocytes, macrophages, and microglia, which mediate damage to the myelin sheath, the protective covering of neurons and axons, leading to disrupted nerve signaling and eventual neurodegeneration [196,197]. Recent researches have emphasized the significant influence of dietary habits and lifestyle on the course of MS, either exacerbating or alleviating symptoms by modulating the systemic inflammatory status, with particular relevance to both relapsing-remitting MS (RRMS) and primary-progressive MS (PPMS) [198,199]. Diets rich in sugar can lead to metabolic dysfunctions, such as insulin resistance, obesity, and systemic inflammation, as well as altering the composition of the commensal gut microbiota, all of which play critical roles in immune function and inflammation [200,201]. Animal studies in experimental autoimmune encephalomyelitis (EAE) mouse model, a widely recognized model for MS, have been demonstrated that diets high in glucose and sucrose can significantly exacerbate inflammation in the brain and spinal cord and worsen the disease progression [35,36]. Mechanically, high-sugar diets can promote the differentiation of CD4^+^ T cells into Th17 cells, a subset of proinflammatory T helper cells, through both direct high-sugar circumstances and sugar-induced alterations in gut microbiota composition, thereby driving the autoreactive T cells attack myelin, further fueling neuroinflammation and demyelination in MS [35,36]. In addition, the breakdown of the BBB, commonly observed in populations on high-sugar diets [102,103], also is a critical early event in the MS progression, allowing autoreactive immune cells to enter the CNS and attack myelin [202]. And high-sugar diet-induced gut dysbiosis can increase the permeability of the gut, allowing bacterial endotoxins, such as LPS to enter the circulation and cross the BBB, where they aggravate neuroinflammation in MS [202,203]. Recent animal studies have also demonstrated that high-glucose environments can exacerbate oligodendrocyte loss [204,205], the cells responsible for producing and maintaining myelin. Chronic hyperglycemia impairs oligodendrocyte progenitor cell (OPC) differentiation and disrupts remyelination, thereby hindering the repair of damaged myelin, worsening neurodegenerative outcomes in MS models.

NMO differs from MS in its underlying pathology, as it is primarily driven by the presence of aquaporin-4 (AQP4) antibodies that target astrocytes, leading to inflammation and damage in the optic nerves and spinal cord [206]. Emerging research have suggested that factors amplifying inflammation potentially predispose individuals to NMOSD, a broader category that includes NMO [206]. One study found that for every 10 g daily increase in sugar consumption, the odds of developing NMOSD rise by 72% [207]. Similar to MS, high-sugar diets can exacerbate neuroinflammation in NMO by promoting systemic inflammation through pathways like NF-κB activation, which increases the production of pro-inflammatory cytokines that damage astrocytes and worsen demyelination in the optic nerves and spinal cord. Additionally, high glucose levels have been shown to impair astrocytes function by increasing oxidative stress [207], compromising their ability to support neuronal health and maintain BBB integrity, which is crucial in preventing immune cell infiltration and subsequent tissue damage in NMO. Hyperglycemia-induced oxidative stress may also directly damage AQP4, exacerbating NMO symptoms [208]. Furthermore, chronic hyperglycemia has been linked to alterations in immune cell function, particularly affecting T cells and B cells [209], both of which play central roles in the autoimmune response in NMO. Elevated glucose levels can promote the differentiation of pro-inflammatory Th17 cells and enhance B cell antibody production, including the pathogenic AQP4 antibodies, thereby intensifying the autoimmune attack in NMO [210,211].

AE is an inflammatory disorder of the central nervous system caused by specific autoantibodies targeting neuronal surface or synaptic proteins. Its core pathological mechanism involves the synergistic action of antibody-mediated synaptic dysfunction and disruption of the BBB [193,212]. Based on the characteristic distribution patterns of affected brain regions, AE can be classified into non-limbic encephalitis, where lesions are not primarily confined to medial temporal lobe structures like the hippocampus [213], and limbic encephalitis (LE), a classic and common subtype characterized by pathological changes and clinical manifestations concentrated in limbic system structures, such as the hippocampus and amygdala [214]. Hippocampal injury in AE may occur through a dual mechanism [215,216]. On one hand, antibodies targeting the voltage-gated potassium channel (VGKC) complex can potentially upregulate delayed rectifier potassium channels, leading to neuronal death and apoptosis [217–219]. They may also induce downregulation of neuronal surface receptors, causing synaptic dysfunction, neuronal hyperexcitability [220,221], and severe impairment of long-term potentiation. On the other hand, immune cell infiltration and the release of pro-inflammatory cytokines (e.g. IL-6) increase BBB permeability, enabling pathogenic antibodies to enter the central nervous system and ultimately damage hippocampal structure and function [194,216,222]. Although substantial evidence supports high-sugar dietary patterns in patients with MS or NMOSD, a direct link to AE pathogenesis remains unestablished. However, emerging evidence suggests the VGKC complex proteins may act as key molecular hubs connecting high-sugar diets to AE pathology. Specifically, as demonstrated by Yan et al., chronic high-glucose environments (>50 mM) disrupt potassium homeostasis by upregulating neuronal Kv2.1 and Kv4.2 channels, increasing K^+^ efflux and promoting neuronal death and apoptosis, ultimately leading to cognitive decline [219]. Furthermore, high fructose intake uniquely enhances cellular excitability by increasing intracellular potassium levels and glutamatergic neuronal secretion [223,224]. This highlights the differential susceptibility of AE subtypes to hippocampal injury: Limbic AE, characterized by anti-LGI1/CASPR2 antibodies, may exhibit sensitivity to high-fructose-induced Kv1.1 downregulation (causing neuronal hyperexcitability), clinically manifesting as anterograde amnesia affecting spatial and declarative memory, disorientation, emotional disturbances, and seizures [215]. In contrast, non-limbic AE subtypes (e.g. anti-NMDAR encephalitis) primarily feature reduced expression of Kv2.1 and Kv4.2 under high glucose, decreasing the I A current. This leads to an increased synaptic NR2B/NR2A ratio and enhanced NMDAR activation, associated with psychiatric symptoms like psychosis and catatonia [218,219]. This evidence supports the need for future clinical studies directly investigating the impact of high-sugar diets on AE, which will help elucidate the underlying mechanisms and lay the groundwork for developing more precise AE treatment strategies.

### 3.4. Alzheimer’s disease

Alzheimer’s disease (AD) stands as the leading neurodegenerative disorder and the most prevalent cause of dementia [225]. Characterized by the accumulation of amyloid-beta plaques, neurofibrillary tangles composed of hyperphosphorylated tau, and widespread neuroinflammation, AD results in cognitive decline, memory loss, and eventually a complete loss of independent function [226,227]. While age, genetics (e.g. APP, PSEN1, or PSEN2), and lifestyle factors like physical inactivity have long been recognized as key contributors to AD [228–230], emerging evidence suggests that high-sugar diets may also play a pivotal role in accelerating the onset and progression of the disease through various metabolic and inflammatory mechanisms.

A large prospective cohort study involving 210,832 participants provided compelling evidence that higher absolute sugar intake significantly increased the risk of developing all-cause dementia, including AD [231]. Specifically, the study found that with each increment in daily sugar consumption, the hazard ratio (HR) for all-cause dementia rose to 1.003 (95% confidence interval [CI]: 1.002–1.004, *p* < 0.001), while the HR for AD increased to 1.002 (95% CI: 1.001–1.004, *p* = 0.005) [231]. This finding was confirmed by several other independent team studies demonstrating a correlation between high sugar intake and the incidence of dementia [156,232,233].

High-sugar diets contribute to several pathophysiological processes associated with AD [234]. One of the key mechanisms involves insulin resistance, which has been increasingly implicated in the development of Alzheimer’s pathology [235]. Commonly referred to as ‘type 3 diabetes’, AD shares several pathological characteristics with diabetes, particularly regarding glucose metabolism dysregulation [236,237]. High-sugar diets can induce chronic hyperglycemia and insulin resistance, both of which compromise cerebral glucose metabolism, leading to neuronal dysfunction, synaptic loss, and memory deficits. For instance, rats fed a 10% sucrose solution for 12 weeks exhibited significant spatial memory impairments, correlating with reduced hippocampal synaptic plasticity and insulin receptor signaling [37,238]. These behavioral deficits are accompanied by metabolic disturbances, such as systemic insulin resistance, dyslipidemia, and leptin dysregulation, highlighting a bidirectional relationship between metabolic dysfunction and cognitive decline [38,238]. And in the 5xFAD mouse model, the absence of insulin signaling in astrocytes exacerbates the Alzheimer’s disease-like phenotype by reducing ATP production and glucose metabolism capacity and impairing A*β* uptake [239]. Notably, the 3xTg-AD mouse model and APP/PS1 models, which recapitulate amyloid-*β* plaques, neurofibrillary tangles, and synaptic dysfunction, have been widely employed to investigate the impact of high-sugar diets on AD progression [37,38,240]. Evidence also suggests that high-sugar diets contribute to A*β* plaque formation. Chronically elevated blood glucose levels have been shown to increase the production and deposition of amyloid-beta, exacerbating the plaque accumulation observed in APP/PS1 mouse [37,240]. The mechanisms linking sugar consumption to amyloid pathology are multifaceted. High glucose levels increase AGEs [83–85], which in turn enhance amyloid precursor protein (APP) cleavage into amyloid-beta peptides *via* the activation of key enzymes, such as *β*-secretase [241]. AGEs not only contribute to amyloid plaque formation but also provoke oxidative stress and inflammation, further promoting AD pathology [86,87,242]. High sucrose intake was found to exacerbate tau protein phosphorylation, which is a key process in the formation of neurofibrillary tangles (NFTs) characteristic of AD, in the 3xTg-AD models by upregulating the mTOR signaling pathway in the brain [38]. The study in a T2DM mouse model further identified a direct link between insulin signaling disruption and tau pathology, that NFTs accumulation was observed in the hippocampus [243,244]. Moreover, neuron-specific insulin receptor knockout (NIRKO) mice exhibited tau proteins hyperphosphorylation, which driven by the loss of insulin-mediated activation of PI3-K, leading to a decrease in the phosphorylation of Akt and GSK-3*β* [243]. Ironically, while excessive sugar intake is a known risk factor for AD, high-sugar feeding regimens are frequently used as experimental models to induce AD-like metabolic impairments in rodents [67,245,246]. This paradoxical approach leverages sugar-induced insulin resistance, neuroinflammation, and mitochondrial dysfunction (hallmarks of both metabolic syndrome and AD) explore mechanistic links between diet and neurodegeneration [247,248]. However, such models may oversimplify human pathophysiology, warranting cautious interpretation.

Another critical pathway by which high-sugar diets contribute to AD is through the induction of neuroinflammation. Chronic hyperglycemia and insulin resistance elevate levels of pro-inflammatory cytokines, such as TNF-*α* and IL-6, both of which have been strongly associated with AD progression [68–72]. This inflammation can lead to breakdown of the BBB, a critical early event in AD pathogenesis. High-sugar diets also can contribute to BBB breakdown by increasing oxidative stress [102,103]. The breakdown of BBB permeability not only promotes peripheral immune cells, inflammatory mediators, and potentially toxic substances to enter the brain parenchyma, but also diminishes the capacity of the brain to clear amyloid-beta *via* the glymphatic system [249–251]. Additionally, high-sugar diets disrupt gut microbiota composition, leading to gut dysbiosis [127], systemic inflammation [41,42], and impairment of BBB [101,102], which in turn influences the development of neuroinflammation and cognitive deficits associated with AD [234].

### 3.5. Parkinson’s disease

PD is a progressive neurodegenerative disorder with age-dependent prevalence, affecting ∼1% of individuals aged 60–69 years, and 6.0% of those over 80 years [252,253]. It is characterized by the progressive degeneration of dopaminergic neurons in the substantia nigra, leading to classic motor symptoms, such as tremors, rigidity, and bradykinesia. Interestingly, many PD patients experience significant changes in eating behaviors, including a notable increase in craving for sweets, even before the onset of motor symptoms [254,255]. The underlying cause of these altered dietary preferences remain unclear, but this phenomenon highlight a potentially important link between diet and disease progression.

A leading hypothesis posits that the increased craving for sugary foods in PD patients stems from striatal DA depletion, which is reduced by ∼40% in early-stage PD [256]. This DA deficit originates from the selective degeneration of dopaminergic neurons in the substantia nigra pars compacta (SNpc), with postmortem studies demonstrating 70–80% neuronal loss in the SNpc before motor symptom onset [257]. Sugar consumption raises blood glucose levels, which triggers insulin release [258]. Insulin, in turn, plays a role in modulating DA dynamics by enhancing DA release in the striatum [259], reducing DA degradation by downregulating monoamine oxidase expression, and increasing DA reuptake by boosting dopamine reuptake transporter [260,261]. It is noteworthy that insulin receptors are abundant in the substantia nigra, the brain region most affected by dopaminergic neuron loss in PD [262–264]. Given insulin effects extensive on both peripheral and CNS [265], it is possible that the increased craving for sugar among PD patients may function as a form of ‘self-medication’, providing temporary relief by boosting brain DA levels and alleviating some motor symptoms associated with the disease [266,267].

However, the relationship between sugar intake and PD is intricate and extends beyond the notion of ‘self-medication’. High-sugar diets are associated with metabolic disorders, such as obesity and type 2 diabetes, which in turn contribute to systemic inflammation, insulin resistance, and oxidative stress, factors increasingly recognized as pivotal in the pathogenesis of PD [268,269]. Insulin resistance, a condition that can be exacerbated by high sugar consumption, has been shown to lead to the inactivation of insulin receptors. This inactivation further results in increased proinflammatory cytokines, heightened oxidative stress, and enhanced alpha-synuclein aggregation in rodent models of PD, all of which accelerate neuronal degeneration. Besides, insulin resistance may further impair insulin signaling in the brain and worsen DA depletion in PD patients [268,270]. Research in PD rodent models has demonstrated that depletion of dopaminergic neurons correlates with disrupted insulin signaling and elevated markers of insulin resistance, suggesting a vicious cycle between progressive insulin resistance and dopaminergic neuron loss, potentially accelerating the neurodegenerative process [271].

Moreover, high-sugar diets may impact PD through several mechanisms, including oxidative stress, mitochondrial dysfunction, inflammation, and gut microbiota disruption. Elevated glucose levels can increase the production of ROS, which damage neurons and worsen motor symptoms in PD [272]. Oxidative stress is also strongly suspected to be involved in chronic hyperglycaemia-induced insulin resistance [273]. High-sugar intake also leads to mitochondrial dysfunction, a critical factor in Parkinson’s disease pathology. Mitochondria are essential for cellular energy production, and their dysfunction can lead to energy deficits in neurons, worsening neurodegeneration [274]. For instance, mitochondrial dysfunction-induced H3K27 hyperacetylation has been linked to epigenetic dysregulation in dopaminergic neurons, exacerbating their degeneration [275]. Inflammation is another significant factor influenced by high-sugar diets. As described above, high-sugar diets can promote systemic inflammation and damage the BBB, which can contribute to neuroinflammation in PD, and further exacerbate dopaminergic neuronal loss and contribute to the progression of motor and non-motor symptoms [276]. Additionally, high-sugar diets disrupt gut microbiota, leading to dysbiosis, which increases intestinal permeability and systemic inflammation, potentially worsening neuroinflammation and PD progression. Studies have found alterations in gut microbiota in PD patients [277], such as a lower abundance of SCFA-producing bacteria [277]. And increased sugar intake was associated with a rise in amyloid-producing bacteria, *Klebsiella* [278]. Bacterial amyloids can increase alpha-synuclein production in the gut and its accumulation in the brain, thereby enhancing cerebral inflammation [278,279].

### 3.6. Mental health

The detrimental link between high-sugar diets, diabetes, and compromised mental health extends beyond classical neurodegenerative pathways. Diabetes-associated hyperglycemia correlates with an increased incidence of depression, anxiety, and accelerated cognitive decline, effects partly mediated by neurovascular dysfunction and chronic inflammation [280]. Notably, diabetes moderates the relationship between age and SRH, with diabetic individuals reporting disproportionately worse SRH as they age compared to non-diabetic counterparts [281]. This decline in SRH may reflect underlying neuropathology, including microvascular damage, altered neurogenesis (particularly in the hippocampus), and early neurodegenerative changes in regions governing emotional regulation (e.g. hippocampus, prefrontal cortex) [282–284]. Consequently, poor SRH in diabetic populations could serve as a sentinel marker for subclinical brain aging and heightened vulnerability to neurodegenerative disorders.

Critically, high-sugar diets independently exacerbate the pathogenesis of depression and anxiety disorders. Globally, these conditions affect an estimated 4.4 and 3.6% of the population, respectively [28], and exhibit significant comorbidity, with many patients experiencing symptoms of both concurrently. Depression is characterized by persistent sadness, hopelessness, and anhedonia, while anxiety involves excessive worry, fear, and tension, both substantially impairing quality of life, cognitive function, and overall well-being [28]. Substantial research interest has focused on the role of diet in the onset and progression of these disorders, revealing complex nutrition-mental health relationships [24–27], with high-sugar diets consistently associated with worsened symptoms. For instance, an epidemiological study in China found that individuals with higher daily intake of free sugars exhibited a greater likelihood of experiencing depression and anxiety [285], and preclinical rodent models demonstrate that such diets reduce activity and induce anxiety-like behaviors [286]. Sugar intake disrupts multiple physiological and neurological processes involved in mood regulation, stress response, and cognitive function, positioning dietary modification as a key intervention strategy [36,132].

The adverse mental health effects of high sugar consumption are mediated through several interconnected pathways. Primarily, high-sugar diets cause significant blood glucose fluctuations, which can impair serotonin synthesis and regulation, thereby contributing to depressive symptoms [133]. Additionally, the resulting rapid glycemic spikes and crashes frequently lead to fatigue and irritability, common features of both depression and anxiety [36,132,133]. Furthermore, excessive sugar intake disrupts hypothalamic-pituitary-adrenal (HPA) axis activity [137,138,145], reducing secretion of both ACTH and corticosterone, a dysfunction linked to increased anxiety and depressive symptoms [287]. Concurrently, diets rich in sugar promote systemic inflammation by elevating pro-inflammatory cytokines (e.g. TNF-*α*, IL-6), which can cross the blood-brain barrier and adversely impact brain function, thereby exacerbating mental health disorders [5,288]. Finally, emerging evidence highlights alterations in the gut-brain axis, where high-sugar diets induce gut dysbiosis, subsequently increasing intestinal permeability, promoting systemic inflammation, and altering gut-derived neurotransmitter production, changes increasingly implicated in the pathogenesis of mood disorders [152,153].

## 4. Conclusions

In summary, accumulating evidence robustly establishes high-sugar diets as detrimental modifiable risk factors for central nervous system disorders through multifaceted pathways. Clinically and experimentally supported associations demonstrate that excessive sugar intake exacerbates ischemic stroke and cerebral atherosclerosis by interacting with genetic susceptibility, metabolic dysfunction, and endothelial inflammation. In autoimmune demyelinating diseases (MS and NMO), high-sugar diets unequivocally aggravate neuroinflammation, autoimmune responses, and neurodegeneration. For neurodegenerative conditions, such diets accelerate Alzheimer’s disease progression *via* insulin resistance, amyloid-beta pathology, and tau hyperphosphorylation, while in Parkinson’s disease, they amplify dopaminergic degeneration through oxidative stress and neuroinflammatory cascades. Concurrently, high-sugar consumption contributes to depression and anxiety by disrupting neurotransmitter systems, elevating systemic inflammation, and inducing gut-brain axis dysregulation. Notably, despite established links to these disorders, direct evidence implicating high-sugar diets in AE pathogenesis remains absent, a significant knowledge gap warranting future investigation. This synthesis underscores the imperative for mechanistic research and positions dietary sugar reduction as a strategic, evidence-based intervention for validated CNS conditions.

## Data Availability

Data sharing is not applicable to this article as no new data were created or analyzed in this study.
